# Quantitative CT Patterns and Functional Loss Related to Welding Fume Exposure, While Handling the Confounding Effect of Cigarette Smoking

**DOI:** 10.1155/carj/2406824

**Published:** 2026-05-06

**Authors:** Merve Demirci Atik, Abdullah Taylan, Aylin Çifci, Ahmet Naci Emecen, Naciye Sinem Gezer, Alp Ergör, Eyüp Sabri Uçan

**Affiliations:** ^1^ Department of Occupational Medicine, Faculty of Medicine, Dokuz Eylul University, Izmir, Turkey, deu.edu.tr; ^2^ Department of Radiology, Dokuz Eylul University, Izmir, Turkey, deu.edu.tr; ^3^ Department of Public Health, Epidemiology Subsection, Faculty of Medicine, Dokuz Eylul University, Izmir, Turkey, deu.edu.tr; ^4^ Department of Public Health, Faculty of Medicine, Dokuz Eylul University, Izmir, Turkey, deu.edu.tr; ^5^ Department of Pulmonary Medicine, Dokuz Eylul University, Izmir, Turkey, deu.edu.tr

**Keywords:** histogram analysis, pneumoconiosis, pulmonary function test, quantitative CT, smoking, welding fume

## Abstract

**Introduction:**

Ill‐defined centrilobular nodules, the most common radiological sign of welding fume (WF) exposure, are also frequently seen in smoking‐related respiratory bronchiolitis due to their common pathophysiological features. Thus, smoking is often a confounding factor in the diagnosis of welder’s pneumoconiosis. This study aimed to investigate the quantitative computed tomography (QCT) patterns associated with WF exposure, while accounting for the effects of cigarette smoking. Additionally, the relationship between WF exposures and pulmonary function tests (PFTs) was also evaluated as the secondary outcome.

**Methods:**

This study involved 136 male employees, comprising 66 welders and 70 controls. The WF exposure index was retrospectively estimated by using a semiquantitative exposure assessment, and cigarette pack‐years were noted. Two radiologists, blinded to exposure information, performed QCT analysis, an objective and reproducible method. PFT results were also compared.

**Results:**

A significant decrease in the histogram’s skewness and kurtosis was observed with WF exposure, in contrast to the effects of smoking. In multivariate analysis, the percentage of voxels in the range of −949 to −850 HU decreased with WF exposure (*p* = 0.037), whereas the percentage of voxels greater than −750 HU increased significantly. Each incremental WF exposure index was associated with a 0.39% decrease in predicted FEV1% (forced expiratory volume in the first second) when adjusted for smoking.

**Conclusion:**

We identified functional losses and distinct QCT patterns associated with exposure to WF and cigarette smoke, both of which may cause chronic lung inflammation. These findings may offer valuable insights for further research.

**Highlights**
• Diagnosing welder’s pneumoconiosis via radiology is challenging and often underestimated due to the high smoking rates among welders.
• A definitive diagnosis is crucial for early termination of exposure, as well as the worker’s compensation and legal rights.
• We identified the significant differences in histogram patterns of smokers and welders in QCT.
• We also confirmed previous studies that showed functional losses linked to welding fume exposure.

## 1. Introduction

Welding fume (WF) contains various particles, including iron, nickel, manganese, silicon, beryllium, nitrogen and ozone [1]. The inflammatory response of lung tissue to these respirable particles can show variations in radiological findings. The most common finding is ill‐defined centrilobular nodules. However, these nodules are not specific to WF exposure; they can also be observed in conditions, such as hypersensitivity pneumonia or respiratory bronchiolitis [1, 2]. Respiratory bronchiolitis, commonly observed in smokers, consists of mild chronic inflammation and the accumulation of pigmented macrophages in the respiratory bronchioles and associated alveoli [3]. Although there may be concurrent mild fibrosis in the walls of the respiratory bronchioles, cases are usually asymptomatic [4]. Smoking habits vary in prevalence from 20% to 60% across different age groups and are particularly common among welders [5, 6]. Due to the confounding effects of smoking, diagnosing welder’s pneumoconiosis through radiology becomes challenging, potentially leading to an underestimation of the attribution to WFs.

Accurate diagnosis of welder’s pneumoconiosis is crucial for early termination of exposure, as well as the patient’s compensation and legal rights. If exposure is limited, welders’ pneumoconiosis can be reversible, whereas prolonged exposure to excessive WFs may cause irreversible fibrosis [7]. The diagnosis of welder’s pneumoconiosis is typically based on an exposure history, lung imaging and the exclusion of other differential diagnoses. Additionally, the demonstration of hemosiderin‐laden macrophages in lung specimens may also indicate exogenous iron oxide accumulation [8]. However, it can also be seen endogenously in pulmonary haemorrhages or interstitial pulmonary diseases of various aetiologies [9, 10]. Increased ferritin concentration in bronchoalveolar lavage fluid or serum, which has been suggested as another diagnostic tool in welder’s pneumoconiosis [6], can also be detected due to smoking [11].

Furthermore, centrilobular nodularity, the primary radiological sign of welder’s pneumoconiosis, may be subjective unless it is severe. It can often be challenging to distinguish between normal and abnormal [1, 12]. Relatedly, interobserver variability in assessing centrilobular nodularity is frequently observed [13]. However, quantitative computed tomography (QCT) is a novel, objective and reproducible method [14]. Many publications investigate the relationship between QCT and lung function in various diseases. It is thought to be useful for follow‐up purposes, especially in fibrotic diseases [14, 15]. In the literature, QCT studies have also been performed on other types of pneumoconiosis, and it has been emphasised that QCT can be a good diagnostic tool due to its objectivity [16].

It is known that both smoking and occupational pollutants may cause increased loss of lung function [17]. Losses exceeding the predicted age‐related decline in forced expiratory volume in the first second (FEV1) of 29 mL/year [18] have been associated with increased morbidity and mortality from chronic obstructive pulmonary disease and cardiovascular disease (approx. 50–90 mL/year) [17]. Therefore, alongside cigarette smoke, it is crucial to quantify the extent of FEV1 loss caused by WFs to monitor welders’ health in the workplace.

This study aims to investigate the impact of exposure to WFs on QCT and respiratory functions while also considering the confounding effect of smoking.

## 2. Methods

### 2.1. Study Population

**FIGURE 1:**
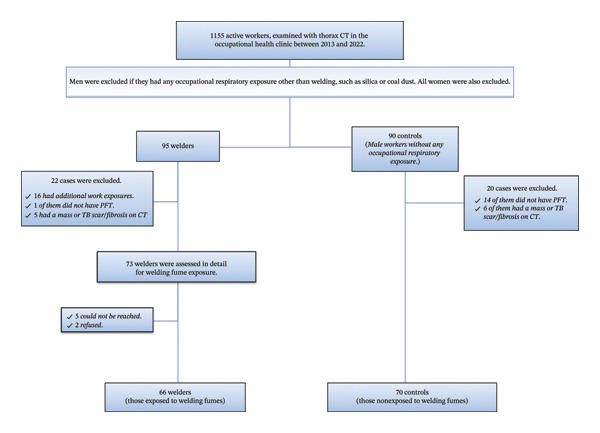
Population flowchart.

The records of 1155 active workers who underwent computed tomography (CT) at the occupational health clinic of Dokuz Eylul University Hospital from 2013 to 2022 were thoroughly reviewed. Among them, 95 welders and 90 controls (male active workers) without occupational respiratory exposure (e.g., silica or coal) were identified. No sampling was conducted in the study; all eligible patients were included. As all welders were male, only males were included in the control group, to account for potential gender‐related differences highlighted in previous studies about histogram analysis [19, 20]. Reasons for CT examinations of the cases included an ILO reading above category 1 during periodic workplace health screenings, as well as working in a dusty industry, even without direct exposure to dust himself or any respiratory complaints. Demographic characteristics and exposure history for all cases were obtained retrospectively from standard exposure information files provided during the hospital admission. Among 90 control cases, six were excluded due to radiological mass/scar/fibrosis or artefact, and fourteen were excluded due to a lack of acceptable pulmonary function test (PFT). On the other hand, of the 95 welders, 16 were excluded due to prior silica exposure, 5 were excluded because of radiological mass or tuberculosis scar/fibrosis, and 1 was excluded due to an insufficiently acceptable PFT. Seventy‐three welders were also contacted by two occupational physicians for a detailed semiquantitative assessment of WF exposure. Five welders could not be reached because their phone numbers were outdated, and two refused. A total of 136 cases, including 66 welders and 70 controls, were involved in the study. The population flowchart for the study is presented in Figure 1. Ethical approval was obtained from the local ethics committee of Dokuz Eylul University (No: 2022/21‐10).

### 2.2. The Semiquantitative WF Exposure Assessment

**FIGURE 2:**
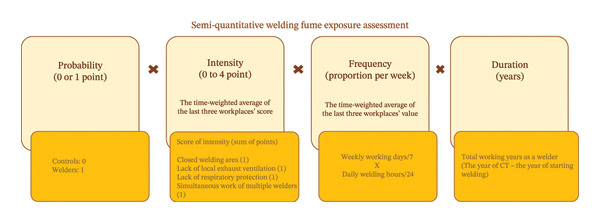
The details of the semiquantitative assessment of welding fume exposure. Footnotes: The total welding fume index was calculated by multiplying the scores of four variables for each case: probability, intensity, frequency and duration. The probability of welding fume exposure was set to ‘1’ for welders, while it was ‘0’ for controls. The variables of intensity and frequency were calculated using the time‐weighted averages of welders’ last three workplaces. Intensity was estimated by summing the scores, assigning 1 point for each variable if it was present. Frequency was determined by multiplying the number of working days per week by the daily working hours. The duration refers to the total number of years worked as a welder.

The method developed by the researchers is similar to studies in the literature [21]. According to this method, the formula ‘probability × intensity × frequency × duration’ was used to determine the cumulative WF index. The probability of WF exposure was set to ‘1’ for welders, while it was set to ‘zero’ for control cases. Therefore, the cumulative exposure index was zero in the control cases, as expected. Intensity was estimated by summing the scores (0 or 1) from variables, such as the enclosed working area, lack of exhaust ventilation, use of respiratory protection and concurrent weld activities. Frequency was determined by multiplying the number of working days per week (out of 7) by the daily working hours (out of 24). As welders are likely to work at more than one workplace during their careers, time‐weighted averages of intensity and frequency were calculated based on the welders’ last three workplaces. Finally, the duration was determined by subtracting the year in which their welding career began from the year the CT was taken. Details of the semiquantitative assessment method are presented in Figure 2.

In the study, organic dust exposure (poultry farming, aviculture, etc.) and any cigarette smoke exposure were recorded in detail. The smoking index was calculated as the ‘number of cigarettes smoked per day/20 × years spent smoking’ (pack‐year). If there was a history of avian exposure, it was recorded as ‘year of exposure’. Smoking index and avian exposure were included in the analysis as confounding variables.

### 2.3. Quantitative CT Evaluation

Chest CT examinations were performed on a 64‐slice CT scanner (Philips Brilliance 64; Philips Medical Systems, Best, The Netherlands) without intravenous contrast. All images were acquired in a supine position with the patient holding his breath during deep‐end inspiration. Scanning parameters were as follows: voltage 120 kV, current 110 mA and slice thickness of 0.9 mm. CT histogram measurements were performed by using licensed imaging analysis software (Myrian; Intrasense, France). The analysis was performed by AT (21 years of experience) and supervised by NSG (24 years of experience). Radiologists were blinded to exposure information. The region of interest (ROI) was determined by drawing contours of the lung area at the level of the spinous process of the 4th thoracic vertebra, as the upper lobes of the lungs are mostly affected by pneumoconiosis. Area histogram analysis was performed manually by segmenting the right and left lung sections separately in the parenchyma window of both lung upper lobes. The lower and upper limits of voxel (the smallest element of a 3‐dimensional image) attenuations measured in this study were determined between (−) 1000 and (+) 100 Hounsfield Units (HU). Mean lung attenuation (MLA), kurtosis (how sharply peaked the histogram is) and skewness (histogram asymmetry) of the HU distribution histogram of all voxels in the selected lung slice of each case were taken as the main dependent variables of the study. For the regression analyses, attenuation values between (−) 1000 and (+) 100 HU were divided into 11 attenuation groups (HU 1–11). The percentage of each attenuation group (voxel%) for each patient’s lungs was taken as a dependent variable.

### 2.4. PFTs

Measurements were performed using a Jaeger Master Screen spirometer by a single technician, in accordance with the relevant guidelines [22]. FEV1 and forced vital capacity (FVC) values from at least 3 acceptable measurements of each patient were automatically selected by the device. FEV1, FVC, FEV1/FVC and forced expiratory flow between 25% and 75% of vital capacity (FEF25‐75) values were reported. Percentages of predicted values (% predicted) calculated by the device based on gender, age, height and race variables were used in the statistics.

### 2.5. Statistical Analysis

Categorical variables were presented as numbers and percentages (%), while continuous variables were expressed as mean ± standard deviation (mean ± SD). We used the Pearson chi‐square test or Fisher’s exact test to compare categorical data. According to normality, continuous variables were compared with Student’s *t*‐test or Mann–Whitney U test between welders and the control group. The study population was divided into four groups to compare histogram patterns according to smoking and WF exposure. The Kruskal–Wallis test and post hoc analyses were performed. We used univariate linear regression analyses to identify the association between the WF index and histogram parameters. Additionally, the effects of the smoking index (pack‐years) and avian exposure (years) were adjusted for in the multivariate analysis. Lastly, multivariate linear regression models were built for PFTs. WF index, smoking pack‐years and avian exposure years were included in multivariable models. Double‐sided *p*‐values of less than 0.05 were considered significant. Statistical analysis was performed with R Version 4.3.1 (A language and environment for statistical computing. R Foundation for Statistical Computing, Vienna, Austria. https://www.R-project.org/).

## 3. Results

The mean ages of welders and controls were 40.86 (±7.415) and 42.59 (±8.214) years, respectively. Except for 14 cases of asthma, there were no other chronic respiratory diseases. Seven of the asthma cases were welders, and the other seven were controls. There was no significant difference in respiratory function test results between the two groups, except for FEF 25‐75 values. More than half of the welders (51.5%) and 61.4% of the controls were current smokers. The mean career years of welders were 21.37 (±10.073) years. In total, 26 cases had long‐term exposure to avians, and environmental asbestos exposure was also identified in 7 cases. There was no statistical difference in the environmental exposures between groups (Table 1).

**TABLE 1:** Features of welding fume exposure groups.

	Control	Welder	*p*‐value
	*n = *70	*n = *66	
Age, mean (SD) (years)	42.59 (±8.214)	40.86 (±7.415)	0.202
Body mass index, mean (SD) (kg/m^2^)	27.74 (±4.224)	27.16 (±3.513)	0.389
Smoking index, mean (SD), pack‐years	15.26 (±13.948)	13.77 (±11.658)	0.503
History of smoking, *n* (%)			0.381
Never	11 (15.7)	10 (15.2)	
Quit	16 (22.9)	22 (33.3)	
Current	43 (61.4)	34 (51.5)	
Years of welding, mean (SD)	0.00 (±0.000)	21.37 (±10.073)	< 0.001
Welding fume index, mean (SD)	0.00 (±0.000)	13.67 (±8.544)	< 0.001
Exposure to avians, *n* (%)			0.114
Yes	17 (24.3)	9 (13.6)	
No	53 (75.7)	57 (86.4)	
Presence of environmental asbestos exposure, *n* (%)			0.713
Yes	3 (4.3)	4 (6.1)	
No	67 (95.7)	62 (93.9)	
FEV1 value, mean (SD) (mL)	3470.00 (±777)	3266.21 (±730)	0.118
FEV1% predicted, mean (SD) (%)	92.10 (±15.531)	82.02 (±17.222)	0.073
FVC value, mean (SD) (mL)	4344.14 (±782)	4102.03 (±894)	0.095
FVC % predicted, mean (SD) (%)	93.64 (±14.453)	91.23 (±13.740)	0.320
FEV1/FVC ratio, mean (SD) (%)	79.78 (±8.373)	77.92 (±8.583)	0.204
FEF 25‐75 flow rate, mean (SD) (L/s)	5.92 (±2.872)	3.25 (±1.266)	< 0.001
FEF 25‐75, % predicted, mean (SD) (%)	86.17 (±22.829)	75.29 (±28.273)	0.015
MLA, median [min, max]	−842.18 [−708, −913]	−842.11 [−669, −909]	0.948^∗^
Skewness, median [min, max]	2.26 [0.63, 3.38]	2.08 [0.40, 3.25]	0.021^∗^
Kurtosis, median [min, max]	6.82 [1.79, 13.47]	5.99 [1.67, 12.82]	0.029^∗^
Occupations, *n* (%)			< 0.001
Managers	7 (10.0)	0 (0.0)	
Professionals	4 (5.8)	0 (0.0)	
Technicians	5 (7.1)	0 (0.0)	
Clerical support workers	2 (2.9)	0 (0.0)	
Service and sales workers	8 (11.4)	0 (0.0)	
Drivers	11 (15.7)	0 (0.0)	
Cleaners and helpers	14 (20.0)	0 (0.0)	
Security officer	10 (14.3)	0 (0.0)	
Labourers in transport	9 (12.9)	0 (0.0)	
Welders	0 (0.0)	66 (100)	

*Note:* Students’ *t*‐test and chi‐square test results of the two main groups.

Abbreviations: FEF 25–75, forced expiratory flow between 25% and 75% of vital capacity; FEV1, forced expiratory volume in the first second; FVC, forced vital capacity; MLA, mean lung attenuation; SD, standard deviation.

^∗^Mann–Whitney U test results are presented.

**FIGURE 3:**
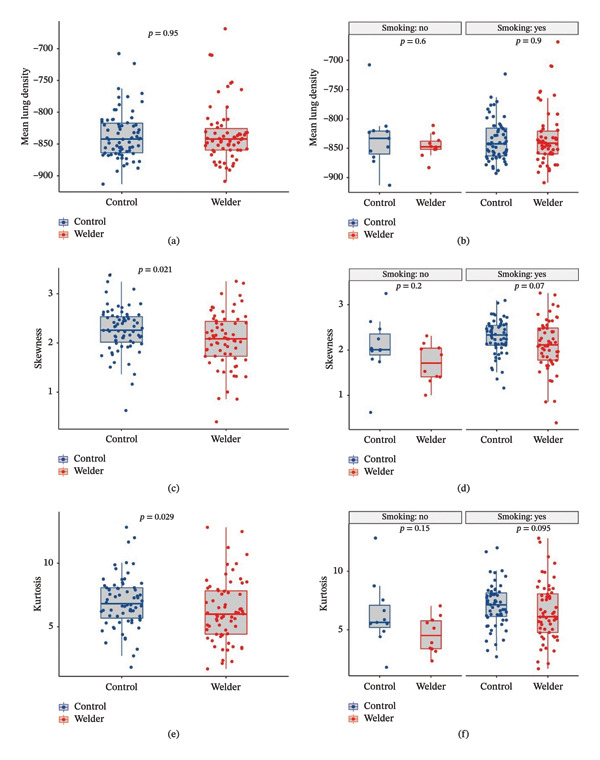
The comparison of QCT parameters between welders and controls, along with the subgroup analysis results.

The skewness and kurtosis of the histogram analyses, the main outcomes of the study, were significantly different between welders and controls according to the Mann–Whitney U test (*p*‐values were 0.021 and 0.029, respectively). Then, the groups were stratified by smoking status, and the difference between groups lost statistical significance (Figure 3). The study population was divided into four groups based on exposure to cigarette and WFs (none, only WFs, only cigarette smoke and both exposed). The four exposure groups showed significant differences in skewness (*p* = 0.0045) and kurtosis (*p* = 0.0042). Post hoc analyses revealed a significant difference between those exposed only to WFs and those who smoked only. Histogram curves and Kruskal–Wallis test results are presented in Figure 4.

FIGURE 4(a) An explanatory schematisation of the quantitative CT (histogram analysis) method. (b) Histogram curves of the four distinct exposure groups. (c) The Kruskal–Wallis test results of the groups. The significance values have been adjusted by Bonferroni correction for multiple tests.

**Figure:**
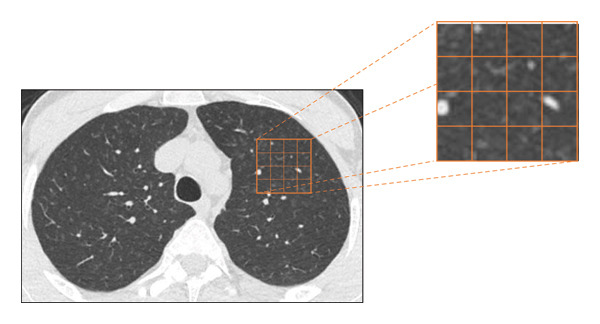
(a)

**Figure:**
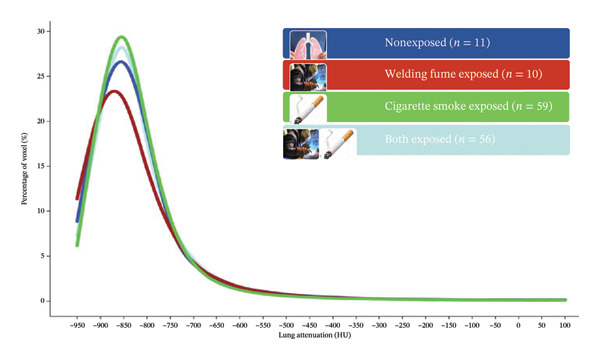
(b)

**Figure:**
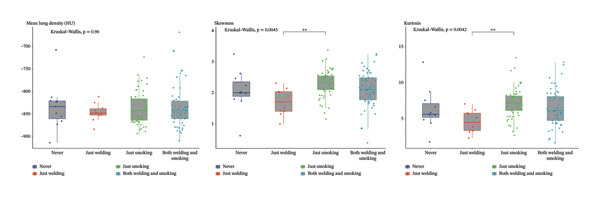
(c)

The frequency of each attenuation group (HU1‐11) was evaluated separately in the linear regression model to assess its relationship with the WF index. Additionally, the effects of smoking and avian exposure were adjusted in the multivariate analysis. In both univariate and multivariate analyses, decreases ranging from −949 to −850 HU and increases from −749 to −450 HU were significant. Moreover, a significant negative association was observed between the WF index and the values of skewness and kurtosis. The linear regression analysis of histogram parameters according to WF exposure is presented in Table 2.

**TABLE 2:** Univariate and multivariate linear regression results between the welding fume index and histogram parameters.

Dependent variables	Univariate analysis		Multivariate analysis	
	β coefficient (95% CI)	*p*‐value	Adjusted β coefficient (95% CI)	*p*‐value
MLA	0.554 (−0.228, 1.316)	0.166	0.539 (−0.239, 1.316)	0.173
Skewness	−0.011 (−0.021, −0.002)	*0.024*	−0.011 (−0.021, −0.002)	*0.020*
Kurtosis	−0.044 (−0.088, −0.001)	*0.047*	−0.045 (−0.089, −0.001)	*0.043*
HU1 (%)	0.000 (−0.001, 0.001)	0.625	0.000 (−0.001, 0.002)	0.539
HU2 (%)	−0.003 (−0.006, 0.000)	*0.041*	−0.003 (−0.006, 0.000)	*0.037*
HU3 (%)	−0.000 (−0.002, 0.002)^∗^	0.976	0.000 (−0.002, 0.002)^∗^	1.000
HU4 (%)	0.001 (0.000, 0.002)	*0.022*	0.001 (0.000, 0.002)	*0.024*
HU5 (%)	0.000 (0.000, 0.001)	*0.017*	0.000 (0.000, 0.001)	*0.017*
HU6 (%)	0.000 (0.000, 0.000)	*0.036*	0.000 (0.000, 0.000)	*0.034*
HU7 (%)	0.000 (0.000, 0.000)^∗^	0.080	0.000 (0.000, 0.000)^∗^	0.075
HU8 (%)	0.000 (0.000, 0.000)^∗^	0.094	0.000 (0.000, 0.000)^∗^	0.090
HU9 (%)	0.000 (0.000, 0.000)^∗^	0.136	0.000 (0.000, 0.000)^∗^	0.131
HU10 (%)	0.000 (0.000, 0.000)^∗^	0.124	0.000 (0.000, 0.000)^∗^	0.117
HU11 (%)	0.000 (0.000, 0.000)^∗^	0.301	0.000 (0.000, 0.000)^∗^	0.281

*Note:* Each row represents a different linear regression model, with histogram parameters as the dependent variables. Univariate models included only the welding fume index as an independent variable. HU1: −1000 to −950, HU2: −949 to −850, HU3: −849 to −750, HU4: −749 to −650, HU5: −649 to −550, HU6: −549 to −450, HU7: −449 to −350, HU8: −349 to −250, HU9: −249 to −150, HU10: −149 to −50, HU11: −49 to +100. In multivariate models, β coefficients for the welding fume index were adjusted for the smoking index (pack‐years) and avian exposure (years). Italicised values represent *p* < 0.05.

Abbreviations: HU, Hounsfield unit; MLA, mean lung attenuation.

^∗^β coefficients are very close to 0.

Regression analysis also indicated a negative relationship between the attenuation areas less than −950 HU and cigarette exposure (*p* = 0.014). However, there was an increasing trend (46%, 49% and 50%, respectively) observed in the entire group’s classification of never, low and high smoking exposure (0, ≤ 20 pack‐years and > 20 pack‐years) in the low‐density areas (LDA, the range of −949 to −850 HU).

The study also evaluated the association between exposure indices and PFTs using multiple linear regression analyses. Each incremental increase in the WF exposure index was associated with a reduction of 0.39% in predicted FEV1. Additionally, the WF exposure index was found to be significantly associated with FEF25‐75, which is known to be potentially more sensitive for the functional assessment of the peripheral airways. Details are presented in Table 3.

**TABLE 3:** Results of the multiple linear regression analysis of the PFTs.

	β Coefficient	Std error	Standardised beta	95% CI	*p*‐value
*Dependent variable: FEV1, % predicted*					
(Constant)	97.333	2.247		92.888, 101.779	< 0.001
Welding fume index	−0.389	0.148	−0.213	−0.682, −0.095	0.010
Smoking (pack‐years)	−0.302	0.104	−0.235	−0.508, −0.095	0.005
Avian exposure (years)	−0.619	0.346	−0.146	−1.302, 0.065	0.076

*Dependent variable: FVC, % predicted*					
(Constant)	96.964	1.993		93.020, 100.907	< 0.001
Welding fume index	−0.244	0.131	−0.157	−0.504, −0.016	0.065
Smoking (pack‐years)	−1.167	0.093	−0.153	−0.351, −0.016	0.073
Avian exposure (years)	−0.367	0.307	−0.101	−0.974, 0.239	0.233

*Dependent variable: FEV1/FVC*					
(Constant)	82.255	1.181		79.919, 84.591	< 0.001
Welding fume index	−0.121	0.078	−0.129	−0.275, 0.033	0.122
Smoking (pack‐years)	−0.156	0.055	−0.235	−0.264, −0.047	0.005
Avian exposure (years)	−0.260	0.182	−0.119	−0.620, 0.099	0.154

*Dependent variable: FEF25-75, % predicted*					
(Constant)	93.090	3.559		−86.050, 100.130	< 0.001
Welding fume index	−0.658	0.235	−0.229	−1.123, −0.194	0.006
Smoking (pack‐years)	−0.490	0.165	−0.242	−0.818, −0.163	0.004
Avian exposure (years)	−0.590	0.547	−0.088	−1.673, 0.493	0.283

Abbreviations: BMI, body mass index; CI, confidence interval; FEF 25–75, forced expiratory flow between 25% and 75% of vital capacity; FEV1, forced expiratory volume in the first second; FVC, forced vital capacity.

## 4. Discussion

According to the results, exposure to WFs decreased the skewness and kurtosis of the histogram curve, contrary to the effects of smoking. This decrease was significant in both univariate and multivariate regression models. In our study, there was a significant negative relationship between cumulative WF exposure and the frequency of LDAs (a range of −949 to −850 HU). This relationship has also been previously described with exposure to other pneumoconiosis agents. Similar to our results, Liu et al. have shown a decrease in the frequency values between the −983 and −778 HU regions with the increasing ILO stage (in the 0, I, II and III stages of coal miners’ pneumoconiosis, 87.31%, 80.51%, 75.27% and 72.99%, respectively) [16]. In contrast to the LDA, the frequency of the ranges −740 and −450 HU showed a positive association with WF exposure in our study. This range above −750 HU has previously been recommended to predict ground‐glass opacities (GGO) in some studies. For example, Kauczor et al. defined the range for GGO as −750 to −300 HU, while Kim et al. defined it as −740 to −174 HU [23, 24]. Based on this background, we can conclude that WF exposure is likely to cause a minimal increase in GGO (ranging from −750 to −450 HU).

Small airway disease, one of the early findings of smoking in the lung, is defined according to the Fleischner Society’s classification of COPD subtypes as the absence of emphysema, along with evidence of physiological obstruction or air trapping on expiratory QCT (rate > 20% at −856 HU) [12, 25]. Even though we used inspiratory CT scans in our study, we obtained significant results concerning the smoking index. We detected an increase in the frequency of LDA (ranging from −949 to −850 HU) and a decrease in areas below −950 HU, which can predict quantitative emphysema. We also observed that the frequency of LDA (−949 to −850 HU) increases gradually with the smoking index (for never smokers, ≤ 20 pack‐year smokers and > 20 pack‐year smokers, the rates are 46%, 49% and 52%, respectively). The findings may be associated with small airway disease in smokers.

Quantitative emphysema is defined in the literature as more than 5% of the lung volume occupied by the attenuation areas ≤ −950 HU on inspiratory CT [19, 26]. However, the findings in the literature are inconsistent regarding the relationship between quantitative emphysema and smoking among smokers without COPD. Grydeland et al. did not find any significant increase in the < 950 HU% areas among non‐COPD smokers related to smoking, which contrasts with the findings for COPD patients [20]. Additionally, some studies have observed discrepancies in emphysema prediction between visual assessment and QCT. In a study, Amaza et al. reported that visual‐only emphysema, rather than quantitative emphysema, was associated with each 10‐pack‐year increase in smoking intensity [19]. The common theory among other researchers with similar findings is that smoking‐related inflammation may mask emphysema and artificially lower the measured lung density decrease, while, visually, the emphysema can still be noticeable [19, 25]. Similarly, in our study, we observed a decrease in the percentage of < −950 HU attenuation with increasing pack‐years of smoking. Although these results may seem contrary to the expectation of smoking‐related emphysema, they can be interpreted as indicating that our cases remained healthy (not with COPD). Moreover, according to the Fleischner Society definition, emphysema is not expected in the small airway disease subtype [12].

According to our results, each incremental WF index was associated with a 0.38% decrease in FEV1 (predicted %). On the other hand, a 0.30% decrease in FEV1 was significantly associated with each pack‐year increase in smoking, as well. Previous studies have also reported greater FEV1 loss among welders than in the reference population, but the overall difference is not notably significant [27–29]. In a meta‐analysis of longitudinal cohort studies, Szram et al. reported a weighted mean difference of −9 mL/year in annual FEV1 decline between welders and reference subjects (95% CI: −22.5 to 4.5, *p* = 0.193). Stratified analysis revealed that the annual weighted mean difference in FEV1 for smokers between welders and the reference group was −13.7 mL/year (95% CI −33.6 to 6.3, *p* = 0.179). In contrast, for nonsmokers, the difference was −3.8 mL/year (95% CI −20.2 to 12.6, *p* = 0.650) [27]. Subsequently, Pouryaghoub et al. also demonstrated the simultaneous effect of WFs and cigarette smoking on respiratory function [30]. Overall, the evidence indicates a greater effect in welders who smoke; therefore, it is necessary to prioritise workplace smoking‐cessation strategies as well as occupational safety measures.

Our study found no significant decrease in the FEV1/FVC ratio associated with increased WF exposure. Data regarding the FEV1/FVC ratio and WF exposure in the literature are also inconsistent [28, 29, 31]. It is stated that this may be linked to the concurrent decrease in FVC, and thus, restriction might also be involved. [27]. Besides, the FEV1/FVC ratio is better known to reflect large‐airway obstruction. However, small respirable particles are more prone to accumulate in the small airways for physiological reasons, and pathological changes in this region occur first [32]. Therefore, we also used FEF 25–75, a commonly used parameter to assess the small airways. We found that FEF 25–75 decreased significantly with both WFs and smoking exposure. This finding might be important to detect early clues.

The first strength of this study was its thorough consideration of various control measures, including closed areas, exhaust ventilation and respiratory protection during the evaluation. The second one, unlike cross‐sectional studies based on snapshot exposure measurements, was a semiquantitative cumulative exposure assessment that considered welders’ lifelong exposure. Because the pathophysiological changes emerge as the result of cumulative exposure, instantaneous measurements may not be adequate for projecting lifelong exposure in different workplace environments. Lastly, potential confounding factors have been controlled by excluding any radiological mass, scar or fibrosis from both groups, and by including participants of the same gender (male), similar age and who had not been exposed to any other occupational respiratory hazards. One of the major limitations of the study was the semiquantitative, retrospective design of the exposure assessment, which may cause recall bias and subjectivity. Another, due to the limited number of cases, statistical significance may have been close to the null, particularly within stratified groups. The loss of significance of histogram differences after stratification by smoking status is thought to be related to this effect. Further studies with larger sample sizes are needed to validate this view. Finally, including only male participants may restrict the generalisability of the findings.

In conclusion, both WFs and cigarette smoking can lead to chronic inflammation and functional loss in the lungs. However, more comprehensive studies are needed to distinguish these similar effects. This will help avoid underdiagnosis of pneumoconiosis, which is a barrier to preventing progression and securing legal compensation, especially among welders who smoke.

## Author Contributions

All authors contributed to the initiation of the research. Merve Demirci Atik, Aylin Çifci and Alp Ergör defined the conceptualisation and methodology. Merve Demirci Atik and Aylin Çifci collected data. Abdullah Taylan and Naciye Sinem Gezer conducted radiological measurements. Merve Demirci Atik and Ahmet Naci Emecen performed statistical analyses. Merve Demirci Atik wrote the article. Alp Ergör, Eyüp Sabri Uçan and Naciye Sinem Gezer supervised. All authors contributed literature research, critically reviewed the final draft and approved it.

## Funding

No financial support has been received for this article.

## Conflicts of Interest

The authors declare no conflicts of interest.

## Data Availability

The data that support the findings of this study are available from the corresponding author upon reasonable request.
